# Vocabulary Learning During Reading: Benefits of Contextual Inferences Versus Retrieval Opportunities

**DOI:** 10.1111/cogs.13135

**Published:** 2022-04-18

**Authors:** Gesa S. E. van den Broek, Eva Wesseling, Linske Huijssen, Maj Lettink, Tamara van Gog

**Affiliations:** ^1^ Department of Education Utrecht University

**Keywords:** Retrieval practice, Testing effect, Effective study strategies, Foreign vocabulary, Contextualized word learning, Incidental word learning

## Abstract

Retrieval practice of isolated words (e.g., with flashcards) enhances foreign vocabulary learning. However, vocabulary is often encountered in context. We investigated whether retrieval opportunities also enhance contextualized word learning. In two within‐subjects experiments, participants encoded 24 foreign words and then read a story to further strengthen word knowledge. The story contained eight target words in a *retrieval context*, which required participants to recall word meaning from memory to understand the text (e.g., “*She borrowed a* ***knyga*** *”*), and eight target words in an *inference context* from which meaning could be *inferred* (e.g., “*She read a* ***knyga*** *”* [book]). After 1 to 2 days, a posttest measured word retention. Reading the words in either the retrieval or inference context increased retention, compared to control words not included in the story. Moreover, in Experiment 1, retention was significantly higher in the inference than in the retrieval condition. In Experiment 2, in which encoding before reading was more extensive and feedback was available, no differences in retention were found between the inference and retrieval + feedback condition (both increased retention, compared to control words). Overall, the findings suggest that the benefits of retrieval may be less pronounced during incidental, contextualized learning than during intentional exercises and that retrieval success must be considered when adding retrieval opportunities to contextualized learning. Under low retrieval success, the better comprehension afforded by an informative context may outweigh the benefits of retrieval opportunities (Exp.1). Yet even when retrieval success was enhanced and feedback was added (Exp. 2), retrieval opportunities were only as beneficial as exposure to rich contextual information.

## 1 Vocabulary learning during reading: Benefits of contextual inferences versus retrieval opportunities

Practicing the retrieval of word knowledge from memory, for example, with flash cards or translation exercises, is effective for foreign vocabulary learning (e.g., Barcroft, 2007; Goossens, Camp, Verkoeijen, & Tabbers, 2014). The benefits of retrieval practice are typically studied with paradigms in which students practice isolated words or phrases (e.g., Carrier & Pashler, 1992; Karpicke & Roediger, 2008). However, vocabulary acquisition occurs to an important extent in context, as learners strengthen their knowledge of words during meaning‐focused activities like reading (e.g., Batterink & Neville, 2011; Elgort et al., 2018). The present study investigated whether such word learning in context would also benefit from opportunities for retrieval.

### 1.1 Word learning through retrieval

Memory retrieval is the act of bringing previously learned knowledge from memory to mind. Practicing the retrieval of words from memory enhances later recall of word form and meaning, compared to other learning strategies, such as studying words with translations (e.g., Karpicke & Roediger, 2008). This advantage of retrieval practice, compared to studying, is known as the *testing effect* (Roediger & Butler, 2011; Roediger & Karpicke, 2006). The testing effect is well‐established in memory research (overview studies in, e.g., Adesope et al., 2017; Dunlosky et al., 2013; Karpicke, 2017; Rowland, 2014) and has been documented with different participant populations and materials, including multiple studies with foreign language vocabulary (e.g., Barcroft, 2007; Carpenter et al., 2008; Carrier & Pashler, 1992; Goossens, Camp, Verkoeijen, & Tabbers, 2014; Karpicke & Roediger, 2008; Karpicke & Smith, 2012; Pyc & Rawson, 2010; van den Broek et al., 2014; see also Nakata, 2016, 2017).

Different explanations have been proposed for the benefits of retrieval practice (overviews in Karpicke, 2017; Rickard & Pan, 2018; Rowland, 2014; van den Broek et al., 2016). Put briefly, these accounts propose that target information in memory becomes more easily retrievable after learners have repeatedly engaged in an effortful, successful mental search for the to‐be‐retrieved information among competing memories. Typically, this higher retrievability is attributed to the selective strengthening of associations between relevant cues (e.g., a foreign word form) and to‐be‐retrieved target information (e.g., the correct word meaning), for example, due to semantic elaboration (Carpenter, 2011; Carpenter & Delosh, 2006) or repeated reinstatement of prior encoding and/or retrieval episodes (Karpicke, 2017; Rickard & Pan, 2018).

In vocabulary learning, retrieval has frequently been studied as part of intentional translation exercises during which learners are aware that the exercises are meant to test or strengthen their word knowledge. For instance, learners were explicitly prompted to retrieve previously encoded words from memory and had to write the words down (e.g., Goossens, Camp, Verkoeijen, Tabbers, et al., 2014), type the words in (e.g., Karpicke & Roediger, 2008), or say the words out loud (e.g., Goossens, Camp, Verkoeijen, & Tabbers, 2014).

Intentional retrieval exercises (e.g., vocabulary translation exercises) are markedly different from more incidental word learning in context, in which improved word knowledge comes as a by‐product of comprehension‐focused processing (e.g., Hulstijn, 2001).^1^ Whereas intentional retrieval exercises have been shown to have a positive effect on word retention, it is unclear whether positive effects of retrieval also occur during more incidental, contextualized learning activities, like story reading. There are two studies that have shown that *adding* (intentional) retrieval exercises may enhance contextualized word learning (Barcroft, 2015; Hulme & Rodd, 2021). Barcroft (2015) found that learners who read a text in their L2 under incidental learning conditions (i.e., without explicit instruction to learn new words from reading) benefited from retrieval opportunities created by replacing target words in the text with their L1 translation and a blank space as a prompt to fill in the missing (foreign) word. Moreover, Hulme and Rodd (2021) showed in a multi‐experiment study that an immediate test (i.e., retrieval practice) after reading narrative stories markedly improved the retention of new word meanings that were introduced in the story (Hulme & Rodd, 2021).

Retrieval exercises can thus enhance contextualized word learning. However, in the studies by Barcroft (2015) and Hulme and Rodd (2021), retrieval practice was still intentional in the sense that participants were explicitly asked to recall the target words and to complete a cloze task, type in a translation or select a translation in multiple‐choice questions. The question addressed in the present study is whether less intentional retrieval, without an overt response or instruction to retrieve words from memory, would also increase retention. To study this question, we build on the results of Van den Broek et al. (2018), who proposed that retrieval can be evoked through a manipulation of the context in which words appear. In a series of experiments, learners in the study by Van den Broek et al. first encoded foreign language vocabulary and then practiced the words further in a translation exercise with either uninformative sentences that contained few contextual clues about word meaning and required memory retrieval to be understood (*retrieval context*: “*I need the* funguo.”) or informative sentences from which word meaning could be inferred (*inference context: “I want to unlock the door. I need the* funguo.”; *funguo* = key). The retrieval sentences led to better learning outcomes: Compared to the inference sentences, retrieval sentences enhanced later recall of word form and meaning as well as comprehension of words in a new context.

Based on the findings by van den Broek et al. (2018), the present study tests whether learners would similarly benefit from uninformative sentences that trigger retrieval of word knowledge when the sentences are embedded in a story that learners read for comprehension. We propose that such a context manipulation during reading could activate the same cognitive mechanisms involved in intentional retrieval exercises—learners processing a newly learned foreign word form and retrieving that word's meaning from memory, thereby strengthening word representations in memory. In this way, retrieval opportunities could be a promising way to enhance the otherwise slow incidental learning of words from context.

### 1.2 Word learning from context

Word learning during reading is a slow process (Laufer, 2003). It typically requires multiple repetitions (e.g., Elgort & Warren, 2014; Hulme et al., 2019; Pellicer‐Sánchez & Schmitt, 2010; Waring & Takaki, 2003), and even when learners understand words in context, they cannot necessarily recall word form or meaning after reading (Haastrup, 1991; Laufer, 2003; Mondria, 2003; Pressley et al., 1987). When contrasted with intentional exercises, incidental learning from context is usually considered less efficient in terms of word retention (e.g., Barcroft, 2009; Laufer, 2005; Paribakht & Wesche, 1996). However, it is also “widely acknowledged that classroom time is typically far too restricted to provide sufficient opportunities for intentional word learning” (Eckerth & Tavakoli, 2012, p. 228). Therefore, researchers seek to identify conditions that increase the chance that learners will retain word knowledge while reading. Factors that have been studied in the past include the number of exposures (e.g., Chen & Truscott, 2010; Hulme et al., 2019; Waring & Takaki, 2003; Webb, 2007), visual enhancement of words in the text (e.g., Han et al., 2008; Kim, 2006), and the amount and variability of information provided in the context (e.g., Bolger et al., 2008; Mondria & Wit‐de Boer, 1991; Webb, 2008). Interventions also often combine word learning from reading with activities that explicitly draw attention to the target word meaning (e.g., Biemiller & Boote, 2006) or form (e.g., Elgort et al., 2016). In general, it is considered beneficial for word learning to increase readers’ elaboration of the word form (i.e., orthography, phonology) and meaning (e.g., Laufer & Hulstijn, 2001; Nation, 2001; Schmitt, 2008). Learners who notice a target word during reading, correctly understand its meaning and contextual use and reinforce the form‐meaning connection through repetition or elaboration are thought to be most likely to remember the word over time (Peters et al., 2009).

Engaging learners in retrieval processes can strengthen the retention of form‐meaning connections over time (Fraser, 1999; Nation, 2001). It is not a given, however, that retrieval opportunities during reading are beneficial if they are created by reducing contextual information (as done by van den Broek et al., 2018). Indeed, constructing a text in such a way that readers can only understand the text if they retrieve word knowledge from memory is at odds with long‐standing claims that learners should be exposed to rich, informative contextual information (e.g., Beck et al., 1983; Stahl & Fairbanks, 1986). For one, contextual information is thought to be beneficial because it allows learners to infer the meaning of words that they do not know. This is considered a form of deep processing that results in better word retention than looking up or being provided with the word meaning (e.g., Carpenter et al., 2012; Grace, 1998; Haastrup, 1991; Hu & Nassaji, 2012).^2^ Second, contextual information is also considered beneficial because processing a word together with relevant, related information could enrich the word representation in memory by creating additional semantic associations and thereby strengthen retention (Schouten‐van Parreren, 1989). In line with this, some studies have reported better learning outcomes for words practiced in context than words practiced without context (e.g., Baleghizadeh & Shahry, 2011) as well as for words studied in a more varied or more informative context than for words studied in a less informative or repetitive context (Bolger et al., 2008; Webb, 2008).

On the other hand, previous memory studies have shown that retrieval practice can produce superior learning when compared to other study techniques that stimulate semantic elaboration (e.g., Karpicke & Blunt, 2011) or that easily give away word meaning (van den Broek et al., 2021). Moreover, not all studies advocate presenting words with rich contextual information. For instance, Mondria and Wit‐de Boer (1991) showed that learners who practiced foreign vocabulary in information‐rich sentences were less able to recall the word form on a posttest than learners who practiced the words in sentences that were less informative. The authors argued that learners’ attention may have been focused on the context rather than the target word (form) in the information‐rich condition, resulting in weak associations between the word form and meaning. This has led others to argue that “quick and easy guessing” may work against the retention of words (Laufer, 2003, p. 572).

Overall, it is clear that exposure to words in context plays an important role in word learning, even though incidental learning from context can be a slow and sometimes unreliable process (e.g., Laufer, 2003; Nagy et al., 1987). The retention of words encountered in context may depend on the degree to which learners pay attention to and deeply process both a word's form and meaning. In some cases, reducing contextual information can trigger such deeper processing of words (e.g., Mondria & Wit‐de Boer, 1991; van den Broek et al., 2018). This makes it plausible that retrieval opportunities can enhance word learning during reading even if they are created by reducing contextual information.

### 1.3 The present study

The aim of this study was to investigate whether newly learned words are remembered better over time if they are, after initial encoding, encountered in a retrieval context that triggers retrieval of form‐meaning associations from memory, compared to an informative inference context from which word meaning can be derived. We expected that reading a story that contains target words would enhance word retention, compared to not encountering the words in a story context, and that a retrieval context would enhance retention more than an inference context (cf. results found with single‐sentence exercises by van den Broek et al., 2018).

These predictions are based on a rich literature showing that retrieval of word knowledge from memory is beneficial for retention (e.g., Karpicke, 2017; Roediger & Butler, 2011; Rowland, 2014) and recent reports that retrieval may be triggered when words are presented in an uninformative sentence context (van den Broek et al., 2018). More specifically, van den Broek et al. (2018) showed that exercises with single sentences were more effective when the sentences created a retrieval opportunity than when they allowed learners to infer word meaning from context. We tested whether such a retrieval manipulation is also beneficial when the retrieval sentences are embedded in a longer story that learners read for comprehension. Compared to single‐sentence contexts (as used by Mondria & Wit‐de Boer, 1991; van den Broek et al., 2018), learners may show different reading strategies and reduced processing of specific words when reading longer, continuous text (Wochna & Juhasz, 2013). In addition, learners may sometimes ignore unfamiliar or difficult words while reading (Nagy et al., 1987). Therefore, it is not a given that encountering words in an uninformative retrieval context during reading will result in an attempt to retrieve word knowledge from memory. However, if retrieval does occur, we expect it to be beneficial for word learning even when learners will not make an overt response. This is because both overt and covert retrieval practice have been found to be beneficial for long‐term retention (Jönsson et al., 2014; Putnam & Roediger, 2013; Smith et al., 2013; Sundqvist et al., 2017; see also Tauber et al., 2018).^3^

## 2 Experiment 1

### 2.1 Method

#### 2.1.1 Participants

A total of 106 Dutch secondary education students took part in the experiment.^4^ Because five students were absent during the second session, data from 101 students were analyzed (*M*_age_ *=* 14.8 years, *SD*_age_ = 0.7, 45 boys/54 girls/2 students who did not fill in their gender). Students were in the third year (cf. U.S. Grade 9) of senior general secondary education or preuniversity education (the Netherlands has a tracked system for secondary education; the participating students were enrolled in the two highest tracks [“havo/vwo”], which prepare for higher education at a university of applied sciences or a research university).

#### 2.1.2 Design

This study had a within‐subject design with three practice conditions: inference condition (initial encoding + reading words in informative story context), retrieval condition (initial encoding + reading words in uninformative story context), and control condition (initial encoding only). The dependent variable was recall accuracy on a delayed retention test, 1 or 2 days later.

#### 2.1.3 Materials

**Fig. 1:**
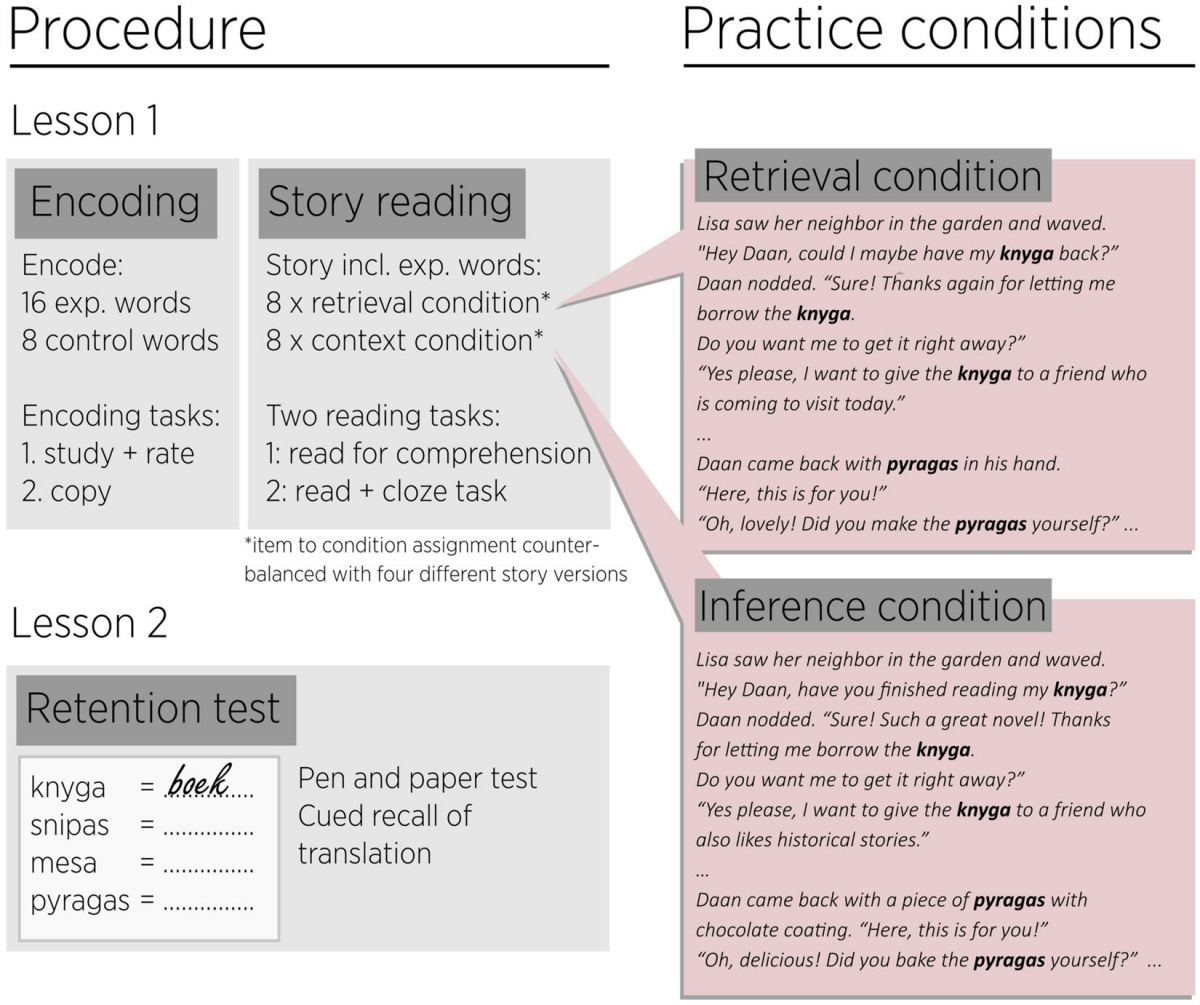
Experimental procedure of Experiment 1 (left panel) and translated, shortened extract of the story as presented in retrieval condition and inference condition (right panel).

Stimuli: The participants studied 24 concrete Lithuanian words such as “*knyga*” (book) or “*pyragas*” (cake). None of the participants reported prior knowledge of Lithuanian. The 24 words consisted of 16 experimental words that were included in story reading and eight control words that were not included in story reading (see Fig. 1; online Supplementary Materials I for a full list of the stimuli). Control words and experimental words were similar to each other in terms of frequency of the Dutch meaning (based on the BasiLex corpus for Dutch school children; Tellings et al., 2014), imageability (derived from Van Loon‐Vervoorn, 1985), and length (*M*_exp =_ 6.5 letters/2.75 syllables; *M*_control_ = 8.1 letters/3.1 syllables). These word characteristics were included in the statistical analyses to control for variation between stimuli.

##### 2.1.3.1 Initial encoding

The experiment began with two encoding assignments. For the first assignment, students were instructed to carefully read a list of the 24 Lithuanian–Dutch word pairs (16 experimental/eight control stimuli) to remember the meaning of the Lithuanian words. In addition, students responded, for each word, to the item “I find remembering the meaning of this word…” on a 5‐point Likert scale from (1) “*very easy*” to (5) “*very difficult*.” This measure of *perceived difficulty* was adopted from the encoding procedure by van den Broek et al. (2018). For the second assignment, students were given the same vocabulary list again (both word‐form and translation), with some letters omitted to create a cloze task. Students were instructed to fill in the missing letters (e.g., they saw “*kepejas = bakery, kepe_ _s = ba_er_”* and filled in the missing letters). The purpose of the encoding exercises was to stimulate the students to actively process the words without engaging in retrieval practice that could cover up the effects of retrieval during the subsequent story reading task. Therefore, the students always saw the complete word pairs during encoding.

##### 2.1.3.2 Story reading with inference and retrieval context

After initial encoding, students were re‐exposed to the 16 experimental words in a Dutch story (length: 16 paragraphs; 1376/1443/1441/1378 words for the four counterbalancing versions, see below). The story was manipulated per word to either be informative and allow readers to infer word meaning (*inference condition*) or to be uninformative and require memory retrieval to understand the target word (*retrieval condition*). The inference condition included strong semantic clues about the meaning of the target word (e.g., “Hey Daan, have you finished reading my **knyga**?”—“Sure, such a great novel, thank you for letting me borrow it!”), whereas the retrieval condition did not (e.g., “Hey Daan, could I have my **knyga** back?”—“Sure, thank you for letting me borrow it.”; see Fig. 1 for a longer extract). In all conditions, definite articles referring to target words were replaced with the word “as” in the text, in order to avoid providing information about the word meaning (as in Dutch, the definite article contains information on the gender of the noun). Target words were printed in bold.

Four versions of the story were made for counterbalancing: Within each story version, eight target words were presented in an inference context and eight in a retrieval context, such that each word appeared in two story versions in the inference context and in two story versions in the retrieval context (see online Supplementary Materials II and III for the exact distribution of words across conditions and the complete stories). The eight control words were chosen in such a way that multiple word meanings were possible for the target words presented in retrieval sentences. For example, to complement the sentence “I work for the *kepejas”* (bakery), the control word *mesininkas* (butchery) was included, as both “bakery” and “butchery” fit the retrieval context. This was done to ensure that students could not simply rely on recalling the only profession that they had encoded prior to reading but needed to retrieve specific word knowledge to understand the story.

To check whether word meaning could indeed be easily inferred in the inference condition and not easily guessed in the retrieval condition, a test of the inference and retrieval versions of each paragraph of the story was conducted with 31 Dutch adults without prior knowledge of the target words. They read each paragraph separately (in either the retrieval or inference condition), filled in up to three guesses for possible word meaning per target word, and rated their confidence in the first guess on a scale from 0 (low) to 10 (high). In the inference versions, although they had no prior knowledge of the words, over 75% of the participants filled in the correct meaning of the target word or a closely related synonym on the first guess. In the retrieval versions, less than 25% of the participants filled in the correct meaning on the first guess with confidence (rating > 5/10), except for three words, for which performance in the retrieval condition was comparably high (>30%). All reported analyses were therefore repeated after excluding these three words; the effects reported in the results section were all replicated.

##### 2.1.3.3 Story reading assignments

There were two reading assignments. First, each student silently read one version of the story. The written instructions stated that the purpose of the reading task was to see if the students could already understand a story that contained the newly learned words (i.e., the emphasis was on text comprehension, not word learning). Second, immediately after the initial silent reading, students were given a cloze task with the same story version, in which 32 Dutch words had been deleted and the students filled in the missing Dutch words (e.g., “I get there quite often to buy croissants, but I have …… seen you at *as kepejas*.” [correct response: *never*]). The purpose of this assignment was to stimulate the students to actively read the story a second time while still focusing on text comprehension. In total, each target word occurred 10 times (2 × 5) during the story reading assignments, following recommendations that multiple encounters are needed for word learning from reading (Pellicer‐Sánchez & Schmitt, 2010; Waring & Takaki, 2003).

##### 2.1.3.4 Retention test

The retention test consisted of a printed list of the 24 Lithuanian words (the 16 target words and eight control words intermixed), and students were asked to write down the Dutch translation of each word. The words were presented in a different order than during initial encoding and story reading. Answers were subsequently digitized and automatically scored, whereby spelling errors were counted as correct when they had an edit distance of two or lower (i.e., no more than two letters had to be added or removed to get to the correct answer; *violni* or *violinn* instead of *violin* were counted as correct; cf. van den Broek et al., 2018). During data processing, answers were also scanned for conceptually correct, alternative responses. In almost all trials, participants either filled in the correct translation, the translation of a different target word that was clearly incorrect, or no response. On three test trials, participants filled in the translation “fish” instead of “salmon.” After discussion among the authors, it was decided not to count these answers as correct.

#### 2.1.4 Procedure

For an overview of the procedure, see Fig. 1. The experiment consisted of two sessions, which took place in students’ regular classrooms. During the whole experiment, a teacher and at least one researcher were present. The entire experiment was performed with pen and paper materials. In Session 1, the students first received a printed booklet with the two encoding tasks that they went through individually at their own pace (there was a time limit of 10 min; all students completed the assignments within that time). When they were finished, the students raised their hands, and the researcher collected the encoding booklet and gave the students another booklet to continue with the story reading assignments, which continued until the end of the lesson (circa 30 min; all students completed the assignments within that time). In Session 2, which was 1 or 2 days later, the retention test was administered (again with a time limit of 10 min; all students completed the test within that time).

#### 2.1.5 Data analysis

We compared recall on the retention test between (a) control words and experimental words (i.e., words that appeared only in the initial encoding assignments and words that appeared in initial encoding and story reading); and (b) experimental words practiced in the inference condition and experimental words practiced in the retrieval condition. Comparisons were made with mixed‐effects models with crossed random effects for participants and items (cf. Baayen et al., 2008; Quené & van den Bergh, 2008) using the glmer function in *lme4* (version 1.1‐10), with a binomial distribution to model the dichotomous outcome variable *Retention* (recall accuracy on the retention test). To test the significance of fixed effects, we used *p*‐values calculated with *lmerTest* (version 2.0‐29; cf. Kuznetsova et al., 2017), which uses Satterthwaite's method for approximation. For interpretation of the effects, we report the (aggregated) proportion of correct recall per condition together with the odds ratio.

### 2.2 Results

#### 2.2.1 Does story reading improve retention?

On average, students remembered significantly fewer control words that were only included in the initial encoding phase (*M* = 0.12, *SD* = 0.15) than experimental words that were additionally presented during story reading (*M* = 0.44, *SD* = 0.22), *β* = 2.58, *SE* = 0.52, *p* < .0001, with higher scores on the retention test for the experimental words included in story reading than for the control words (*Odd's ratio (OR)* = 13.2). This effect of story reading remained significant when word frequency, imageability, and perceived difficulty were added to the model to control for possible differences between control words and experimental words (three separate models, all *p*s < .0001 for the effect of condition).

#### 2.2.2 Does a retrieval context lead to better retention than an inference context?

**Fig. 2:**
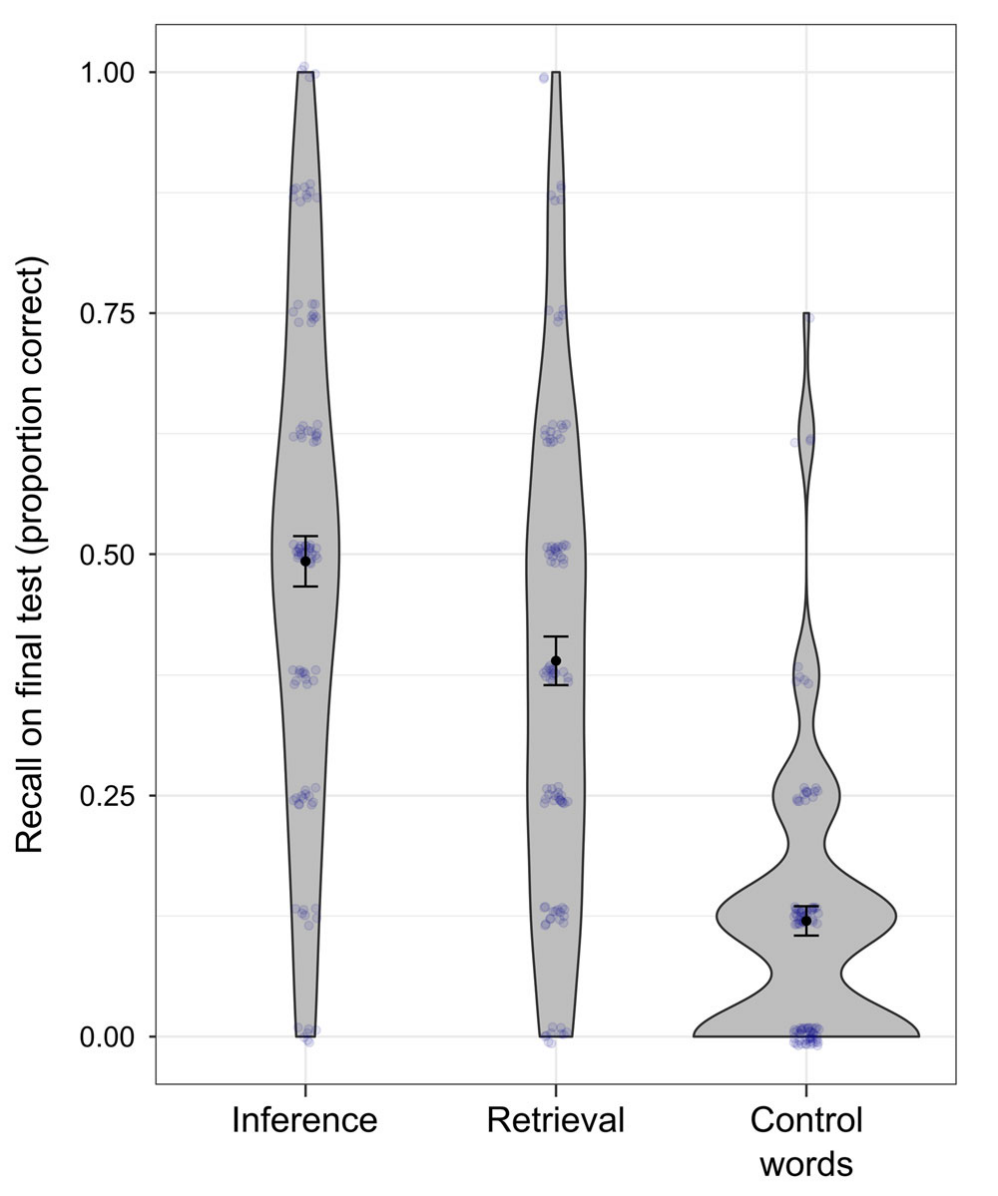
Violin plots of recall performance on the retention test in Session 2 (Experiment 1). *Note*. For this visualization, the proportion correct on the test was aggregated per participant per practice condition; observations were jittered to reduce overplotting.

Contrary to our predictions, students remembered significantly *fewer* words in the retrieval condition (*M* = 0.39, *SD* = 0.25) than in the inference condition (*M* = 0.49, *SD* = 0.26), *β* = −0.62, *SE* = 0.12, *p* < .0001, *OR* = 0.54, see Fig. 2. This main effect of practice condition on word retention remained significant when the word characteristics frequency, imageability, and perceived difficulty were added to the model. It also remained significant when the story version was added to the model to control for counterbalancing versions (the story version did not enhance the model fit, χ(3) = 0.69, *p* = .87; it did not have a significant main effect on word retention nor was there an interaction of practice condition and story version).

#### 2.2.3 Exploratory analysis: Does word difficulty moderate the effect of story context?

**Fig. 3:**
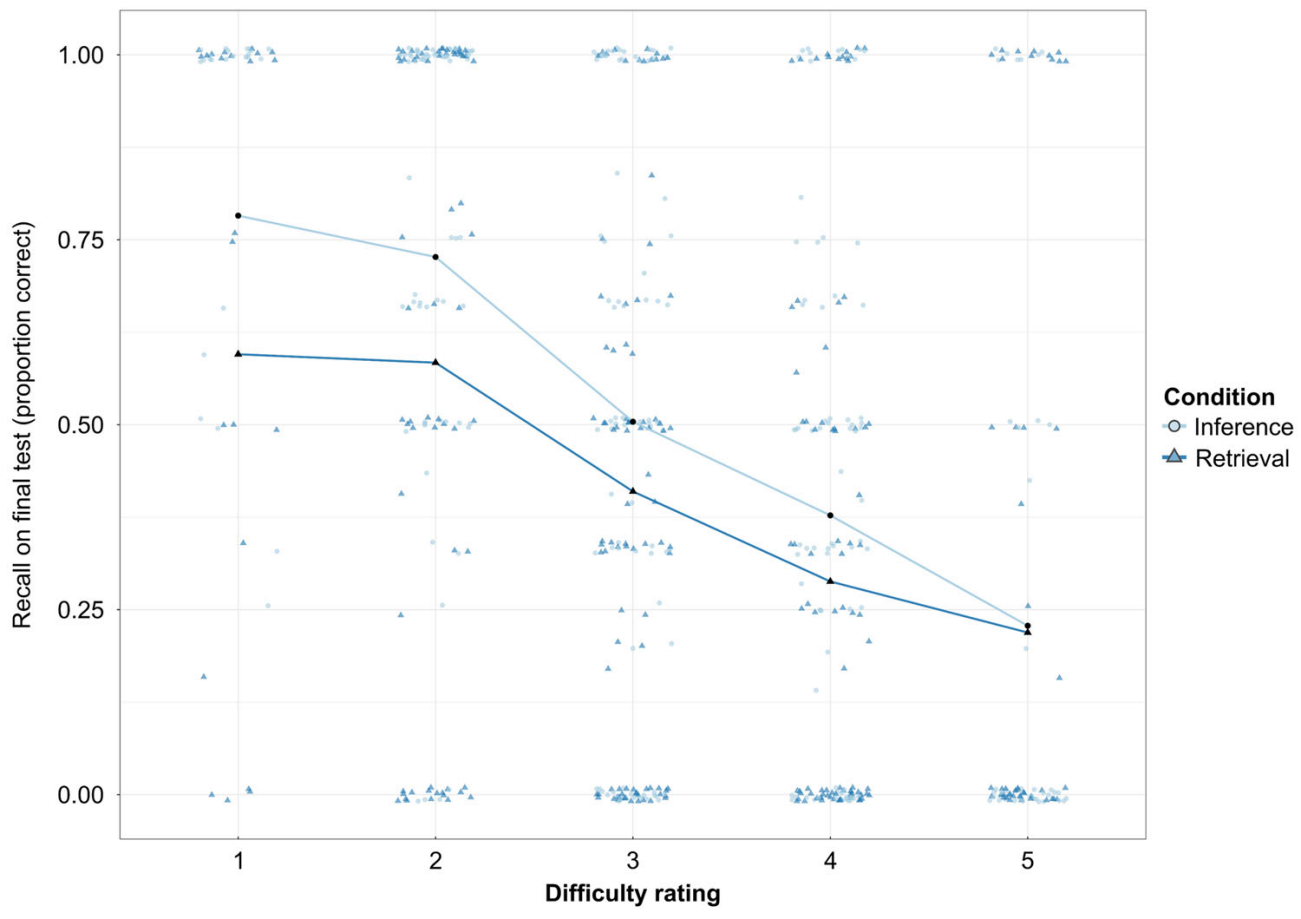
Average proportion of items translated correctly on the retention test, split by difficulty rating (Experiment 1). *Note*. For this visualization, the proportion correct on the test was aggregated per participant per difficulty rating [Scale (1) “*very easy*” to (5) “*very difficult*”] and story condition. Observations are jittered to avoid overplotting (width = 0.2). The black markers indicate the group mean.

Because students made no overt response during reading, it is unknown how many of the target words were understood during reading, especially in the retrieval context in which students had to retrieve word meaning from memory.^5^ However, during initial encoding, students rated how difficult they found it to remember each word, and this rating may give an indication of how difficult it was for students to recall word meaning during the subsequent reading task. Indeed, perceived difficulty negatively predicted recall on the retention test (*β* = −0.58, *SE* = 0.10, *p* < .0001, *OR* = 0.56), with lower recall for words that the student perceived as more difficult. Perceived difficulty also interacted with the story condition (*β* = 0.25, *SE* = 0.12, *p* = .045), with higher retention in the inference condition than in the retrieval condition only for words with difficulty ratings of “1” to “4” but comparable retention in the two story conditions for the words with the highest difficulty rating “5” (see Fig. 3). Overall, the model with story condition and perceived difficulty again showed a main effect of story condition on word retention, with higher recall on the retention test in the inference condition than in the retrieval condition (*β* = −1.41, *SE* = 0.42, *p* = .0007, *OR* = 4.17).

### 2.3 Discussion

In line with our first hypothesis, newly learned words were retained better over time if they were, after initial encoding, repeated in a story context. However, contrary to our predictions, we did not replicate the benefits of retrieval opportunities reported by van den Broek et al. (2018). Instead, the informative story context led to *higher* posttest results than the retrieval context.

It is possible that retrieval opportunities are *less* beneficial or that rich contextual information is *more* beneficial when learners read a long, coherent narrative (as in the present study) than during exercises with isolated, single sentences (used in the previous study by van den Broek et al., 2018). Compared to van den Broek et al.’s and other studies that found no benefits or negative effects of presenting words in an informative context from which word meaning can be inferred (e.g., Mondria, 2003; Mondria & Wit‐de Boer, 1991; Pressley et al., 1987; van den Broek et al., 2018), the to‐be‐learned words were embedded in a richer, more coherent narrative in the present study. This may have provided participants with more distinct or varied contextual details that they could later use to recall word meaning and may have been more beneficial than opportunities for (covert, incidental) memory retrieval created by the uninformative retrieval context.

However, there were also procedural differences in the setups of Experiment 1 and the study by van den Broek et al. (2018), which could explain the differences in findings. One important difference between Experiment 1 and the study by van den Broek et al. (2018) is that the latter provided participants with feedback about the correct word meaning, whereas our story reading task did not include explicit feedback. Memory research has shown that retrieval practice is only beneficial for retention when learners either successfully retrieve the correct answer from memory or when failed retrieval attempts are followed by feedback from which learners can re‐encode the answer (e.g., Rowland & DeLosh, 2015; van den Broek et al., 2014, 2018). If students did not remember a target word in Experiment 1, exposure to the retrieval context was therefore likely not beneficial—students simply encountered an unknown word in an unclear sentence and had no way to understand the word's meaning.^6^ In contrast, exposure to the retrieval context in the previous study by van den Broek et al. (2018) was likely always beneficial because participants received explicit feedback, which allowed them to re‐encode words that they failed to retrieve from memory. This issue of retrieval failure was possibly exacerbated in Experiment 1 by the fact that participants underwent a shorter encoding task prior to the retrieval exercise, compared to the study by van den Broek et al. (2018), making it more likely that participants did not understand some of the words in the retrieval context. In short, van den Broek et al. (2018) used a procedure that made it more likely to find benefits of retrieval practice than Experiment 1. Therefore, we made changes in Experiment 2 to test whether the benefits of the inference condition would hold when the impact of (limited) retrieval success in the retrieval condition was reduced by means of a longer encoding phase and feedback.

## 3 Experiment 2

In Experiment 2, we made two adjustments to reduce the impact of retrieval failure during reading, which should make the retrieval condition more effective. First, we extended the encoding phase prior to reading. The new encoding procedure included the same tasks as those used by van den Broek et al. (2018) and was expected to increase retrieval success during reading. Higher retrieval success during practice typically increases the benefits of retrieval opportunities (Jang et al., 2012; Rowland, 2014). Second, we added feedback to the retrieval condition. Words were presented first in an uninformative retrieval context, followed later in the text (during the final repetition of the word in the story) by an inference context from which word meaning could be derived. In this way, the informative final sentence functioned as a form of (incidental/indirect) feedback that allowed learners to re‐encode words even if they could not initially retrieve the word meaning. This retrieval + feedback condition was expected to resolve the issue of learners not being able to access word meaning in case of retrieval failure, yet without turning the reading task into more explicit word learning. Moreover, the retrieval + feedback condition was expected to be beneficial because learners who notice that they do not understand a word might process that word more deeply when the meaning is later revealed (cf. pretesting effects: Richland et al., 2009; and the “noticing” component of word learning, Nation, 2001). Overall, the procedural adjustments should increase the chance of finding benefits of the retrieval condition in Experiment 2 by increasing retrieval success with longer encoding and reducing the impact of retrieval failure with feedback. Conversely, if the retrieval condition would still produce inferior learning, that would strengthen the conclusion that retrieval opportunities during reading are less beneficial for learning than rich contextual information.

A final change between Experiments 1 and 2 concerns the participant population. Participants in Experiment 1 were adolescents (secondary education students), whereas participants in the study by van den Broek et al. were university students. Whereas retrieval practice is beneficial for learners of different ability levels (Jonsson et al., 2021), inference processes during reading are likely more difficult for younger, less experienced readers (see, e.g., Kuhn & Stahl, 1998). This may have made the inference of word meaning a more effective exercise for the students in Experiment 1 than for the adults who participated in the previous study, as the students possibly engaged in more effortful (yet successful) inferences, which are thought to enhance word retention more than fluent inferences (Hu & Nassaji, 2012, 2014). To test if the benefits of the inference condition replicate across populations, we, therefore, recruited adult participants in Experiment 2, working with a more similar target group as van den Broek et al. (2018), again possibly increasing the chance of finding benefits of retrieval opportunities.

### 3.1 Methods

#### 3.1.1 Participants and design

Experiment 2 had the same within‐subjects design as Experiment 1. Eighty‐three^7^ participants were recruited via prolific, an internet platform for adults to volunteer for online experiments (www.prolific.co). The data of 72 participants could be used for analyses (*M*_age_ = 32.92, *SD* = 10.73; gender: 40 female, 30 male, two other) after the exclusion of five participants who had very short reading times for the self‐paced story reading task (below 3 min in total and/or below 10 s for one or more of the pages) and six participants who did not complete Session 2 within 2 days after Session 1. In separate control analyses, we also excluded 10 additional participants who reported taking notes or covering the screen to test themselves during the experiment. The payment was GBP 5.10 for the full experiment, which took approximately 50 to 60 min in total. Restrictions on the website were set to only recruit participants who reported being fluent in English and not fluent in Lithuanian and who did not reside in Lithuania or neighboring countries according to the website's prescreen survey. We also restricted participation to prolific users who had positive prior experience with online experiments (100% acceptance rate, at least three prior studies). In our own questionnaire during the experiment, none of the participants reported prior knowledge of Lithuanian, the language of the items taught.

#### 3.1.2 Materials and procedure

Experiment 2 was an online experiment that was implemented using gorilla.sc (Anwyl‐Irvine et al., 2020). Settings enforced that participants could only access the experiment using a desktop computer or laptop. Upon reading the study description, participants could immediately sign up and start the experiment. They were asked to take the experiment in a quiet environment. As in Experiment 1, Session 1 comprised initial encoding and the story reading task with a within‐subject manipulation of the context (*inference condition* and *retrieval + feedback condition*), and Session 2 comprised the posttest. Session 2 was made available 2 days after Session 1. The parts of the procedure that were changed in comparison to Experiment 1 are described in the following sections.

##### 3.1.2.1 More extensive initial encoding

The encoding phase in Experiment 2 was extended, compared to Experiment 1. In Experiment 2, we used the same three encoding tasks as van den Broek et al. (2018), which ensured extensive encoding of the word–translation pairs prior to the reading task while not offering any retrieval opportunities. In the first encoding task, participants were instructed to carefully study 24 foreign words (16 experimental/eight control stimuli). The word–translation pairs (Lithuanian target words with English translation) were presented one by one for 8 s; then, a rating scale was added, and participants responded to the question “How difficult is it to remember this word?,” using a 100‐point slider from (0) “*very easy*” to (100) “*very difficult*.” For the second encoding task, participants were asked to think of a “mnemonic association” (a mental image, rhyme, etc.) to help them remember each word and to type in a short description. If participants could not think of a helpful association, they had to type over the word–translation pair twice. For the third encoding task, the word–translation pairs were shown three more times. On each trial, participants first saw one word–translation pair for 1 s and then responded to the question “Have you learned this word?” on a 100‐point slider from (0) “*No, I do not remember the word yet*” to (100) “*Yes, I fully remember the word*.” van den Broek et al. (2018) adapted the number of repetitions during this task to participants’ responses; we instead presented all words three times (the average number of repetitions per word pair reported by van den Broek et al. (2018) to ensure that the number of repetitions was the same for control and experimental words).

##### 3.1.2.2 Story reading in the inference and retrieval + feedback condition

Participants read an English translation^8^ of the story used in Experiment 1. The story was split into eight pages, and participants could navigate back and forth between the pages at their own pace. Each of the 16 target words was included in five sentences within one paragraph as in Experiment 1. The sentences about a specific target word were either all informative and allowed readers to infer word meaning (*inference condition*) or were initially uninformative but ended with an informative sentence (*retrieval + feedback condition*). More specifically, the new *retrieval + feedback* condition combined the first four sentences of the original retrieval condition—which did not reveal the word meaning and created retrieval opportunities—with the fifth sentence of the inference condition. This last sentence was informative so that participants could infer the word meaning. We consider this setup comparable to retrieval with feedback because participants could first attempt to retrieve the word meaning from memory while reading the first four sentences but were then able to infer the word meaning from the last sentence as a form of feedback on their (possible) retrieval attempts.

##### 3.1.2.3 Stimuli

The same 16 experimental words were used as in Experiment 1. Two of the eight control words were changed. One control word (*meziningas* = butcher) was replaced by a different word (*pienininke* = farmer) because the word “butcher” appeared in the translated story. A second control word, *prieziura*, was paired with the (fictive) translation “*art*” because the original translation “*care*” did not fit the translated story (the control word *prieziura* functioned as a competitor of one of the target words). To ensure that these changes had no effect on our conclusions, the analysis comparing the control and experimental words was repeated once with and once without the adjusted control words, showing the same (significant) difference in retention.

##### 3.1.2.4 Posttest

The same scoring procedure was applied as in Experiment 1. Three conceptually correct alternative answers were counted as correct: “bicycle” instead of “bike,” “dance” instead of “dancing,” and “artwork” instead of “art.” These alternative answers did not occur in Experiment 1.

#### 3.1.3 Data analysis

The same analyses were conducted as in Experiment 1. In addition, after obtaining the results, we added a post hoc equivalence test (Lakens et al., 2018) to test whether the nonsignificant difference between the inference condition and the retrieval + feedback condition was small enough to reject the presence of a meaningful effect in the population. Equivalence testing compares an observed effect to the smallest effect size of interest. We derived the smallest effect size from a meta‐analysis of retrieval practice effects (Rowland, 2014) using the lower bound of the confidence interval around the overall effect of retrieval practice compared to other study conditions (*g* = 0.42^9^). This is a more conservative estimate than the mean effect size (which was *g* = 0.54; cf. Perugini, Gallucci, & Costantini, 2014, as cited in Lakens et al., 2018). In addition, we tested whether the condition effect was small enough to reject the presence of even smaller effects than *g* = 0.42 in the population and report the smallest effect size for which the null equivalence hypothesis was rejected. Posttest results were averaged per participant across items before testing for equivalence using the *t_TOST* function (Caldwell, 2022) from the TOSTER package (version 0.4.0) in R.

### 3.2 Results

#### 3.2.1 Does story reading improve retention?

**Fig. 4:**
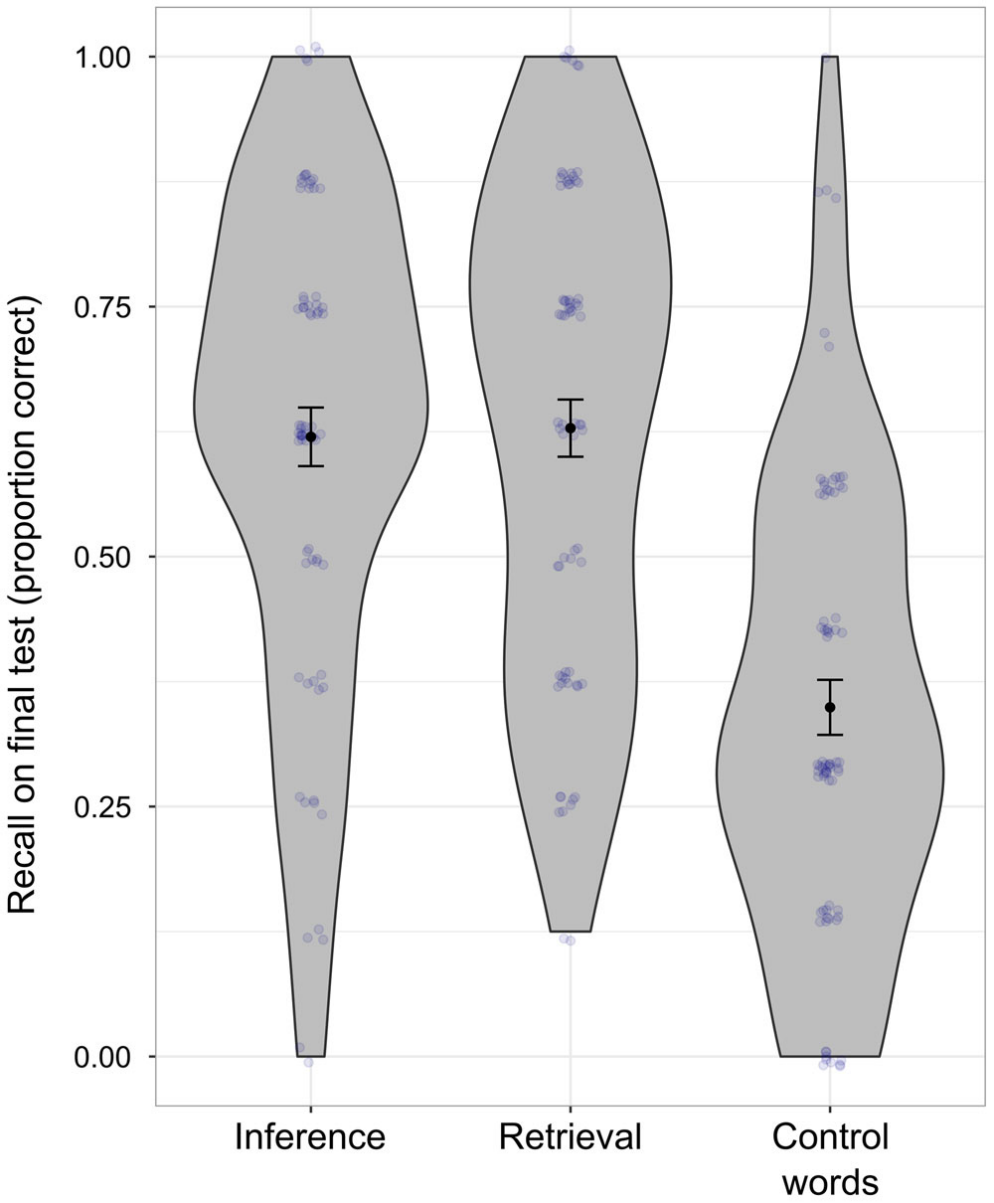
Violin plots of recall performance on the retention test in Session 2 (Experiment 2). *Note*. For the visualization, the proportion correct on the posttest was aggregated per participant per practice condition; observations were jittered to reduce overplotting.

As in Experiment 1, participants remembered significantly fewer control words that were only included in the initial encoding phase (*M* = 0.35, *SD* = 0.23) than experimental words that were additionally presented during story reading (*M* = 0.62, *SD* = 0.22), *β* = 1.54, *SE* = 0.48, *p* = .001, *OR* = 4.64, see also Fig. 4. This effect of story reading on posttest performance remained significant when word frequency, imageability, and perceived difficulty were added to the model to control for possible differences between control words and experimental words (three separate models, all *p* < .05 for the effect of type of word). The effect also remained significant when excluding two control words that were different from Experiment 1 (see Method section), *β* = 1.44, *SE* = 0.46, *p* = .002, *OR* = 4.23, and after excluding the 10 participants who reported taking notes or covering the screen during practice, *β* = 1.66, *SE* = 0.43, *p* = .0001, *OR* = 5.27.

#### 3.2.2 Does a retrieval + feedback context lead to better retention than an inference context?

There was no main effect of the story context on posttest recall, *β* = −0.02, *SE* = 0.14, *p* = .91, *OR* = 0.98, with similar posttest results for the inference condition (*M =* 0.62, *SD* = 0.25) and the retrieval + feedback condition (*M* = 0.63, *SD* = 0.24, see also Fig. 4). The nonsignificant main effect of the story condition was replicated in control analyses that included as additional predictors the story version (counterbalancing condition), word frequency, imageability, or difficulty rating. There was also no significant interaction between story condition and word difficulty: Story condition had no significant effect on posttest recall, independent of how easy or difficult participants rated the words. Finally, the nonsignificant condition effect was also found after excluding 10 participants who reported taking notes or covering the screen during practice, *β* = 0.05, *SE* = 0.16, *p* = .76, *OR* = 1.05.

##### 3.2.2.1 Testing for (the absence) of a meaningful effect

A post hoc equivalence test (see Method section) indicated that the nonsignificant difference between the inference condition and the retrieval + feedback condition was small enough to reject the presence of a meaningful effect in the population, assuming a smallest effect size of interest of *d* = 0.42, *t*(71) = −3.24, *p* < .001. This means that, in the population, the condition effect is likely smaller than *d* = ± 0.42. Further exploratory analyses using an uncorrected alpha level of .05 showed that the condition effect was also statistically equivalent for smaller effect sizes, up to *d* = 0.24, *t*(71) = −1.67, *p* = .0496, which corresponds to a difference in raw scores on the posttest of less than 4.9%.

### 3.3 Discussion

Experiment 2 was set up with the purpose to create conditions that were more similar to the previous study by van den Broek et al. (2018), which made it more likely that we would find benefits of retrieval opportunities during reading. However, despite the adjusted procedure, which led to substantially higher posttest recall scores than in Experiment 1, Experiment 2 did not show significant benefits of the retrieval + feedback context over the inference context. Instead, story reading in both conditions enhanced retention to a similar extent relative to the control words that were not included in the story reading task.

The lack of difference between the two story conditions in Experiment 2—equivalence tests suggest that the difference in retention scores between the two conditions is likely smaller than 4.9% in the population—strengthens the conclusion that, at least in the present setup, creating retrieval opportunities during reading may not enhance word learning compared to the presentation of words in an informative context that made it easy for readers to infer word meaning. This finding, like the results of Experiment 1, differs from the results reported by van den Broek et al. (2018) and from the broader memory literature on retrieval benefits (e.g., Adesope et al., 2017; Dunlosky et al., 2013; Karpicke, 2017; Roediger & Butler, 2011; Rowland, 2014). This raises questions about the use(fulness) of retrieval opportunities outside intentional exercises, which we will address in the General Discussion.

## 4 General discussion

This study investigated whether newly learned words are remembered better over time if they are, after initial encoding, repeated in a story context and whether repetition in a story context that evokes retrieval of form‐meaning associations from memory is more effective than a story context that allows inference of word meaning (cf. results with sentence contexts by van den Broek et al., 2018).

In line with our first hypothesis, repetition in a story context increased word retention: Words presented in either the inference or retrieval context were remembered significantly better than the control words that were not included in story reading in both experiments. This replicates previous findings that exposure to words in context enhances word retention (e.g., Laufer, 2003; Nation, 2015; Schmitt, 2008). Importantly, exposure to words in the uninformative retrieval context also benefited word retention (compared to no exposure). This finding extends previous research in which *intentional* retrieval practice enhanced word retention (e.g., Karpicke & Roediger, 2008; van den Broek et al., 2014) by showing the benefits of retrieval opportunities during *incidental* word learning, where retrieval was not instructed but triggered through a manipulation of the context.

However, contradicting our second hypothesis and findings by van den Broek et al. (2018), the retrieval context was not preferable to the inference context. Participants remembered the meaning of the words encountered in the inference condition either better than the meaning of words encountered in the retrieval condition (when the encoding phase was relatively short; Experiment 1) or equally well in both conditions (when the procedure was adjusted to make the retrieval condition more effective through a longer encoding phase and feedback embedded in the story context; Experiment 2).

As the benefits of retrieval practice, compared to other study strategies, are a robust phenomenon in the memory literature (e.g., Adesope et al., 2017; Dunlosky et al., 2013; Karpicke, 2017; Roediger & Butler, 2011; Rowland, 2014), the lack of retrieval benefits, compared to the inference condition in the present study, was unexpected. Yet it is also interesting, as it could enhance our understanding of possible boundary conditions of retrieval‐enhanced learning, like the retrieval format (overt vs. covert retrieval), retrieval mode (intentional vs. incidental retrieval), and the impact of retrieval success.

### 4.1 Boundary conditions of retrieval‐enhanced learning

#### 4.1.1 Retrieval format and instructions

One important difference between the present study and the previous study in which retrieval opportunities were created by manipulating the context of words (van den Broek et al., 2018) is that we did not require an overt response, whereas participants typed in the word meaning in the previous study. We thus focused on *covert* retrieval, whereas van den Broek et al. (2018) used overt retrieval. Previous studies on covert versus overt retrieval for the practice of pair‐associate learning (e.g., word pairs) found that both types of retrieval are similarly beneficial for retention (Putnam & Roediger, 2013; Smith et al., 2013; in a small meta‐analysis by Sundqvist et al., 2017, the overall effect‐size comparing covert and overt retrieval was close to 0). However, it has been suggested that overt retrieval may encourage learners to give a more precise or exhaustive response when lengthy or complex information must be retrieved, whereas covert retrieval may result in less complete or more superficial retrieval of only part of the information (Tauber et al., 2018). In this way, our covert retrieval paradigm may have encouraged less complete retrieval than the task used by van den Broek et al. (2018). Moreover, we assumed that participants would attempt to retrieve word knowledge from memory when confronted with an unclear reading context (in memory experiments, it has been suggested that retrieval is to some extent automatic and occurs when a cue is presented, e.g., Craik et al., 1996). However, it is possible that participants did not process all target words in this way. Indeed, prior research on incidental word learning from context suggests that readers sometimes fail to recognize when they do not understand a word correctly or ignore unknown words in a text (Laufer, 2003; Nagy et al., 1987).

Retrieval may have also been suboptimal or unreliable in this study because participants were not explicitly instructed to engage in retrieval when encountering the target words in context. One previous multi‐experiment study compared word retention after participants performed a task in *retrieval mode*, where participants intentionally tried to recollect information from memory, and a task in *generation mode*, where participants were asked to produce the first information that came to their mind (Karpicke & Zaromb, 2010). Doing the same task in retrieval mode led to higher retention than the generation mode, even when participants produced the same responses. Whereas participants in the previous study by van den Broek et al. (2018) were in an intentional retrieval mode during the translation exercises, our setup triggered retrieval in a more incidental way. If, because of this, participants were focused on comprehension of the story rather than a conscious recollection of specific words, this may have reduced retrieval‐enhanced learning parallel to the effect observed by Karpicke and Zaromb (2010). Overall, our findings put the question for future research on the agenda of whether the benefits of retrieval are as pronounced when retrieval occurs covertly and incidentally, compared to intentional retrieval exercises with overt responses.

#### 4.1.2 Retrieval success

Another crucial factor in explaining our findings is likely retrieval success during reading. The combined findings of Experiments 1 and 2 suggest that the relative benefits of the inference and the retrieval context depended at least in part on the degree to which participants were able to access word meaning during reading. Especially with limited encoding prior to reading—as in Experiment 1—an uninformative retrieval context can put readers at a disadvantage when they are unable to recall the target words during story reading, whereas they could always infer word meaning from the informative context. In contrast, inference and retrieval conditions became similarly effective when we extended prior encoding and introduced a form of indirect feedback in Experiment 2, measures that increase retrieval success and reduce the impact of retrieval failure (e.g., Rowland & DeLosh, 2015; van den Broek et al., 2014, 2018). It is possible that after even more drastic changes in the encoding procedure, the results might be more in favor of the retrieval condition. However, a retrieval manipulation that requires such extensive prior encoding is of limited practical significance. After all, incidental learning is typically meant to replace rather than supplement extensive intentional learning, and a more straightforward measure would be to use intentional retrieval exercises from the outset.

### 4.2 Limitations and future research

A number of characteristics of the present study need to be taken into account when considering its implications. First, learners read a story written in L1, which contained L2 target words (similar to the approach used by van den Broek et al., 2018, with sentences) in bold font. This somewhat artificial task allowed us to clearly compare the effects of retrieval and inference. Had we used a story in L2, differences in prior knowledge and/or limited comprehension of the L2 would likely have obscured the effects of retrieval and inference. Nevertheless, it might be an interesting avenue for future research to investigate retrieval versus inference effects in an L2 story context.

Second, because of the focus on incidental word learning, learners did not provide overt responses. This made it impossible to measure whether words were correctly recalled when reading the retrieval context or correctly inferred from the inference context. Although we do not know for sure whether participants paid attention to the target words in the text, story reading in both conditions markedly enhanced retention, compared to the control words, both after limited initial encoding in Experiment 1 (*M*_control_ = 0.12; *M*_retrieval_ = 0.39 on posttest) and after more extensive encoding in Experiment 2 (*M*_control_ = 0.34; *M*_retrieval+feedback_ = 0.63). This makes it unlikely that participants ignored all target words or were unable to recall any of the words in the retrieval context. On the contrary, both retrieval and inference contexts must have triggered processing that was beneficial for word retention to explain the consistent, large benefits over control words. Future research using think‐aloud, or eye‐tracking measures could provide further insight into the thought processes involved in reading, however. This could show, for instance, whether the retrieval context was selectively beneficial only for those words for which participants made the effort to retrieve the word meaning from memory and/or when learners managed to infer word meaning from the feedback sentence after a failed retrieval attempt (cf. Halamish & Bjork, 2011; Kornell et al., 2011; van den Broek et al., 2014).

Process measures could also address possible concerns regarding reading strategies in the retrieval + feedback condition in Experiment 2. To stay true to the incidental nature of the story reading task, we included an informative sentence in the text from which participants could infer the word meaning. This form of feedback differs from the typical feedback given in retrieval exercises, where participants first submit an overt response and are then shown the correct answer as explicit feedback. A possible concern regarding our manipulation is that participants may have skipped over the uninformative retrieval sentences to directly infer word meaning from the final, informative sentence containing the target word or may have skipped over the informative sentence and not processed feedback. Process measures could document the extent to which such reading strategies occur. However, the issue of readers skimming ahead is likely reduced in more natural texts that have a less predictable structure than our experimental materials (e.g., because the spacing of sentences about a specific target word varies more across the text). This reduces the chance of undesired reading strategies.

Third, before the story reading task, students encoded only 24 different words. It is possible that this setup influenced the recall processes that occurred in the retrieval condition: Rather than analyzing the foreign word and trying to recall the associated translation, learners may have tried to remember the different word meanings that they encoded previously to search for those words most compatible with the context. This strategy may have limited benefits for the retrieval condition. However, the same could be said about the previous study by van den Broek et al. (2018), which did show the benefits of a retrieval context. Moreover, from a practical point of view, it is likely that language methods include a limited number of new words per unit, which are also included in the accompanying text.

### 4.3 Conclusion

The present study showed that language learners who recently encoded new foreign vocabulary words benefitted from additional exposure to the words during story reading. Although word learning during reading is typically slow (e.g., Laufer, 2003), the 20‐ to 30‐min reading task in the present study had significant benefits for word retention on a posttest several days after reading in both experiments. Story reading was beneficial both when the context was informative and allowed readers to infer the meaning of target words and when the context was uninformative and created retrieval opportunities. However, retrieval success must be considered when adding retrieval opportunities to contextualized learning: When word knowledge from prior encoding was limited, an informative context was more beneficial than a retrieval context (Experiment 1). In contrast, even when the procedure in Experiment 2 was adjusted to optimize conditions for effective retrieval practice by means of extended prior encoding and feedback, the retrieval condition was still only as effective as the inference condition (Experiment 2). These findings highlight potential boundary conditions of retrieval effects in incidental, contextualized word learning, which should be further investigated. That is, although intentional retrieval exercises are beneficial for vocabulary retention (e.g., Karpicke & Roediger, 2008), retrieval that is triggered in a more incidental way and without an overt response may not be preferable over alternative incidental learning tasks such as exposure to rich contextual information. At present, we conclude that the safer approach to word learning during reading is to expose learners to a context that is rich in information than to an uninformative retrieval context, as the richer context was beneficial after limited prior encoding and comparably effective as a retrieval context otherwise.

## Conflict of interest

The authors declare that they have no known conflicts of interest to disclose.

## Notes

1 The distinction between intentional and incidental learning is complex. In this paper, we follow the definition that in intentional learning, learners are instructed to learn target words, whereas in incidental learning, the instruction focuses on comprehension of language input (cf. Huckin & Coady, 1999).

2 Note, however, that empirical support for benefits of inferring word meaning from context is mixed (e.g., Hulstijn, 1992; Mondria, 2003). For example, Mondria (2003) found that when learners made inferences, this increased the total study time but was not more effective than just presenting learners with the word meanings.

3 It is possible that overt retrieval may cause stronger benefits than covert retrieval especially when the to‐be‐retrieved information is complex and overt retrieval is more likely to encourage participants to fully retrieve the answer (Tauber et al., 2018). We come back to this in the general discussion.

4 As there was no comparable prior research, we could not predict an exact effect size for the condition effect. When planning the study, we calculated sample sizes needed to be able to detect a small to medium effect size in repeated‐measures analyses with a power of 0.80, using the G*power software (Faul et al., 2007). This led to estimates between 70 (t test with *d* = 0.3) and 90 participants (Anova with *f* = 0.15). The actual sample size was larger because we accounted for dropout and recruited students in full classes. As we decided to use a mixed model approach for the statistical analyses, statistical power was presumably higher, compared to the analyses of aggregated data on which the power analysis was based (e.g., Baayen et al., 2008).

5 We did not include a direct measure of retrieval success (i.e., overt retrieval tasks) during encoding or reading task, to avoid overwriting potentially more subtle retrieval effects from manipulations of the story context, and in order to keep word learning during reading incidental.

6 Although (lack of) retrieval success is a plausible explanation for the different findings in Experiment 1 and the study by van den Broek et al. (2018), exploratory analyses of Experiment 1 do not fully support this explanation. In particular, we found that the inference context was more beneficial than the retrieval context for all words except the most difficult ones. If difficulty ratings predict how easily students could retrieve word meaning during reading, retrieval success should be lower and benefits of the inference context should be larger for more difficult words (rather than smaller as was observed). However, there was a limited number of recalled items in the highest difficulty category and the relation between self‐reported word difficulty and subsequent retrieval success has not been empirically established. Therefore, these exploratory analyses need to be interpreted with caution.

7 Sample size was based on a power calculation with G*power (version 3.1; Faul et al., 2007). With outcomes set to the values of Experiment 1 (*p* = 49% in the control condition; *OR* = 0.54), the sample size needed for a power of 0.80 is 77 in standard logistic regression. We overrecruited by 10% to account for possible drop‐out during the study.

8 A few adjustments were made when translating the story to English to ensure that the story was understandable for an international audience (e.g., we removed reference to specific Dutch baked goods and well‐known Dutch ice skaters). A pilot with 10 participants from the same prolific population that was also recruited for the main experiment confirmed that participants without prior knowledge of the target words were able to infer the correct word meaning from the informative story context as well as the retrieval + feedback context.

9 For the present study, this corresponds to *d* = 0.4245: Solving formulae (4) and (5) from Rowland (2014) for *d*, and entering the *n* from Experiment 2: *d* = g/J = g/(1 – (3/(4*(*n* – 1) – 1).
