# Supplementary material for: Vocabulary Learning During Reading: Benefits of Contextual Inferences Versus Retrieval Opportunities
Source: Cogn Sci. 2022 Apr 18;46(4):e13135. doi: 10.1111/cogs.13135 (PMC9285746; doi:10.1111/cogs.13135)
Supplement: Supplementary file 1 [file COGS-46-0-s001.docx]

**Online Supplementary Materials**

**I – Complete Vocabulary list**

| **Type of word** | **Lithu-anian** | **Dutch** | **English translation** | **Fre-quency** | **log(freq.)** | **Imagea-bility (Dutch)** | **SD (imageab.)** | **length* (Lith.)** | **length* (Dutch)** |
| --- | --- | --- | --- | --- | --- | --- | --- | --- | --- |
| exp. | apyranke | armband | bracelet | 186 | 2.27 | 6.4 | 0.76 | 8 | 7 |
| exp. | ciuzimas | schaatsen | to iceskate | 417 | 2.62 | 6.43 | 1.14 | 8 | 9 |
| exp. | darbas | huiswerk | homework | 525 | 2.72 | 5.03 | 1.81 | 6 | 8 |
| exp. | gamta | natuur | nature | 1431 | 3.16 | 5.5 | 1.43 | 5 | 6 |
| exp. | gydytojas | dokter | doctor | 1469 | 3.17 | 6.7 | 0.62 | 9 | 6 |
| exp. | kepejas | bakker | baker | 552 | 2.74 | 6.3 | 1.15 | 7 | 6 |
| exp. | knyga | boek | book | 7637 | 3.88 | 6.73 | 0.58 | 5 | 4 |
| exp. | lasisa | zalm | salmon | 57 | 1.76 | 5.63 | 1.63 | 6 | 4 |
| exp. | ledai | ijs | icecream | 1962 | 3.29 | 6.63 | 0.72 | 5 | 3 |
| exp. | mesa | vlees | meat | 1058 | 3.02 | 6.33 | 1.21 | 4 | 5 |
| exp. | plaukti | zwemmen | to swim | 1855 | 3.27 | 6.57 | 0.9 | 7 | 7 |
| exp. | pyragas | taart | cake | 1091 | 3.04 | 5.5 | 1.06 | 7 | 5 |
| exp. | smuikas | viool | violin | 293 | 2.47 | 6.77 | 0.57 | 7 | 5 |
| exp. | snipas | spion | spy | 190 | 2.28 | 4.03 | 1.94 | 6 | 5 |
| exp. | sokiai | dansen | to dance | 1838 | 3.26 | 6.4 | 1.07 | 6 | 6 |
| exp. | ziureti | horloge | watch | 565 | 2.75 | 6.87 | 0.43 | 7 | 7 |
| control | krepsinis | bal | ball | 55 | 1.74 | 6.8 | 0.48 | 9 | 3 |
| control | vairavimo | rijles | driving lesson | 35 | 1.54 | ** | ** | 9 | 6 |
| control | prieziura | zorg | health care | 1089 | 3.04 | 3.6 | 1.75 | 9 | 4 |
| control | mesininkas | slager | butcher | 339 | 2.53 | 6.7 | 0.64 | 10 | 6 |
| control | plaktukas | hamer | hammer | 295 | 2.47 | 6.83 | 0.53 | 9 | 5 |
| control | vedlys | tovenaar | magician | 311 | 2.49 | 5.9 | 1.27 | 6 | 9 |
| control | dviratis | fiets | bike | 2870 | 3.46 | 6.93 | 0.36 | 8 | 5 |
| control | duona | brood | bread | 1779 | 3.25 | 6.67 | 1.06 | 5 | 5 |
|  |  |  |  |  |  |  |  |  |  |
|  |  |  |  |  |  |  |  |  |  |
|  |  | **Average per type of word** | |  |  |  |  |  |  |
|  |  | **Exp.** | **Control** |  |  |  |  |  |  |
| log(frequency) | | 2.9 | 2.6 |  |  | * length = number of letters | | |  |
| Imageability | | 6.1 | 6.2 |  |  | ** freq. and imageability unknown | | | |
| Word length (Lit) | | 6.4 | 8.1 |  |  |  |  |  |  |
| Word length (Nl) | | 5.8 | 5.4 |  |  |  |  |  |  |
|  |  |  |  |  |  |  |  |  |  |

**II – Distribution of Target Words across Inference Condition and Retrieval Condition**

|  | **Story Version** | | | | |
| --- | --- | --- | --- | --- | --- |
|  | **Version 1** | **Version 2** | **Version 3** | **Version 4** | |
| **knyga** | Inf | Retr | Inf | Retr |  |
| **pyragas** | Retr | Inf | Retr | Inf |  |
| **mesa** | Inf | Retr | Inf | Retr |  |
| **lasisa** | Retr | Inf | Retr | Inf |  |
| **snipas** | Inf | Retr | Inf | Retr |  |
| **darbas** | Retr | Inf | Retr | Inf |  |
| **apyranke** | Inf | Retr | Inf | Retr |  |
| **ziureti** | Retr | Inf | Retr | Inf |  |
| **gydytojas** | Inf | Retr | Retr | Inf |  |
| **gamta** | Retr | Inf | Inf | Retr |  |
| **sokiai** | Inf | Retr | Retr | Inf |  |
| **smuikas** | Retr | Inf | Inf | Retr |  |
| **ciuozimas** | Retr | Inf | Inf | Retr |  |
| **plaukti** | Inf | Retr | Retr | Inf |  |
| **ledai** | Inf | Retr | Retr | Inf |  |
| **kepejas** | Retr | Inf | Inf | Retr |  |

*Note. Inf* = Inference condition, *Retr* = Retrieval Condition.

**III. Translated Extract and Complete Story Version 1.**

**Extract**

The table below contains a translated extract of the paragraph about the target word “as **gydytojas**” (*the doctor*). The table was added to visualize corresponding sections of the story in retrieval and inference condition; students read a continuous text for the experiment.

| **Retrieval context (uninformative)** | **Inference context (informative)** |
| --- | --- |
| “Are you going to the university’s open day next week, Daan?” | “Are you going to the university’s open day next week, Daan?” |
| Daan shook his head. “No, I’m already sure what I want to study. I want to be **as gydytojas**.” | Daan shook his head. “No, I'm already sure that I want to study medicine. I want to be **as** **gydytojas**.” |
| “Have you known for a long time that you want to become **as gydytojas**?” | **“**Now I'm trying to imagine you in a white coat with a stethoscope around your neck!” Lisa laughed. “Have you known for a long time that you want to become **as** **gydytojas**?” |
| “Yes, pretty much. When I was a child, I already used to pretend play that I was **as gydytojas**.” | “Yes, pretty much. When I was a child, I wanted to cure everyone with my toy first aid kit. So I used to pretend play that I was **as** **gydytojas**.” |
| “How cute! Your mother is also **as gydytojas**, isn’t she? She is probably very pleased that you will follow in her footsteps!” | “How cute! You mother is a surgeon, right? She is probably very pleased that you will also become **as gydytojas**.” |
| “Yes she is, and my father is too, so working as **as gydytojas** is a family tradition.” | “Yes she is, and my father also works in the hospital, so working as **as gydytojas** is a family tradition.” |

**Story Version 1**

Lisa zag haar buurjongen en zwaaide.
"Hey Daan, heb je mijn **knyga** uitgelezen?” Daan knikte. “Ja, het was een prachtige roman, dankjewel voor het uitlenen van je **knyga**. Zal ik hem gelijk halen?”
“Graag. Mijn vriendin is ook geïnteresseerd in oorlogsverhalen, dus ik wil **as knyga** aan haar geven.” Daan liep het huis in, haalde de bladwijzer tussen de pagina’s vandaan en kwam terug met **as knyga** in zijn ene hand en een **pyragas** in zijn andere hand.
“Hier, een bedankje.”
“Oooh, wat lief, dankjewel voor **as pyragas**! Heb je die **pyragas** zelf gemaakt?”
“Nee, ik heb hem gekocht.”
“Hij ziet er mooi uit. Dat had niet gehoeven!”
“Jawel, echt fijn dat ik je **knyga** mocht lezen!”
“Natuurlijk, jij bedankt voor **as pyragas**! In welke winkel heb je **as pyragas** gekocht?”
“Gewoon in de supermarkt. Daar hebben ze altijd goede aanbiedingen. De hamburgers zijn nu in de aanbieding. Die moet je eens halen. Ze hebben goed **mesa** in die supermarkt!”
Lisa schudde haar hoofd.
“Ik ben vegetariër, dus ik eet nooit **mesa**.”
"Oh, dan moet je **as lasisa** eens proberen."
“Oh goed idee! Ik hou erg van **lasisa**."
“Ik heb een lekker recept met **lasisa**. Zal ik je dat geven?”
“Graag! **Lasisa** ziet er ook mooi uit.”
Daan knikte.
“Ik maak ook graag stoofpot met **mesa** voor gasten, maar aangezien jij als vegetariër geen **mesa** eet, zal ik je dat recept niet geven.”
Lisa knikte. "Nee, het maakt niet uit of het rund, kip of varken is. Ik eet echt geen **mesa**!"
“Haha, komt goed Lisa. Ik stuur het recept naar je door!”
“Dankjewel!”

“Maar ik moet gaan Daan, want ik ga met vrienden naar die actiefilm over die Russische **snipas** die de CIA heeft geïnfiltreerd.”
“Is dat die film waarin die geheim agente undercover gaat om **as snipas** te ontmaskeren?”
“Klopt, en die acteur die de slechte dubbelagent speelt, speelde in de film die wij vorige maand keken ook een **snipas**.” 
Daan lachte. “Inderdaad! Hij ziet er gewoon uit als een James Bond achtig type. Daarom speelt hij zo vaak een **snipas**.”
“Veel plezier! Niet spoilen of de missie van **as snipas** wordt voltooid, hè?”
“Ik houd mijn mond! Doei, Daan!”
“Later, Lisa!”

De volgende dag ging Daans telefoon.
“Hey Daan, met Lisa, heb je zin om vanavond dat recept met **lasisa** eens uit te proberen?”
“Ik kan niet. Ik heb nog **darbas**.”
“Oh jammer."
“Heb je misschien na **as darbas** nog tijd?”
“Ik denk dat het wel even tijd kost. Dus ik denk het niet.”
"Heb jij eigenlijk geen **darbas**?"
"Nee, vandaag niet."
“Vind je het goed als ik ondanks je **darbas** toch even langskom?”
“Prima! Gezellig.”
“Dan kom ik om 5 uur, oké?”
“Oké, dan kunnen we ook best vanavond samen koken. Tot zo!"

Daan was klaar met **as darbas** en daarna kookten ze.
“Het is inderdaad een heerlijk recept Daan! Waar heb je het vandaan?”
“Ik heb het ooit gegeten op vakantie. Trouwens, Lisa, hoe was je verjaardag?”
“Leuk! Ik heb van mijn ouders een paar zilveren oorbellen gekregen met een bijpassende ring, ketting en **apyranke**.”
“Wauw! Ik zie dat je hem nu niet om je pols hebt hangen. Heb je een foto van je **apyranke**?”
Lisa pakte haar telefoon. “Kijk, op deze foto komen de kralen van mijn **apyranke** net onder mijn mouw uit.”
“Mooi hoor. Kan je bedeltjes hangen aan je **apyranke**?”
"Klopt! Ik kan kleine zilveren hangertjes en kralen sparen en wisselen, zodat mijn **apyranke** er steeds uitziet als een ander sieraad.”
“Echt een mooi cadeau!”
“Jij bent toch ook bijna jarig Daan? Wat ga jij vragen?”
“Klopt! Ik heb een nieuw **ziureti** gevraagd.”
“Dat is een goed cadeau! Maar ik heb eigenlijk nooit een **ziureti** nodig.”
“Ik ook niet hoor, maar op school denk ik steeds vaker: had ik nu maar een **ziureti**.”
“Daar heb je een punt. Dat overkomt mij ook vaker, maar toch heb ik nooit een **ziureti** gekocht.”
“Jammer eigenlijk, want het is toch fijn om een **ziureti** te hebben."

“Ga jij volgende week naar de open dag van de universiteit, Daan?”
Daan schudde zijn hoofd. “Nee, ik weet al zeker dat ik geneeskunde wil studeren. Ik wil namelijk **gydytojas** worden.”
“Ik probeer me jou nu voor te stellen in een witte jas met een stethoscoop om je nek!”, lachte Lisa. “Weet je al lang dat je **gydytojas** wil worden?”
“Ja, eigenlijk wel. Ik wilde vroeger al iedereen beter maken met mijn speelgoed verbanddoos. Dus speelde ik al voor **gydytojas**.”
“Wat schattig! Jouw moeder werkt toch ook op de operatiekamer? Die vindt het vast leuk dat jij ook **gydytojas** wordt!”
“Ja, en mijn vader werkt ook in het ziekenhuis, dus het werken als **gydytojas** zit in de familie!"
“Wat grappig! Bij mij is dat niet het geval. Ik wil graag werken in **as gamta**."
“Weet je al wat je precies in **as gamta** wil gaan doen?”
“Nee, ik weet nog niet goed welke specifieke beroepen er zijn, dus ik weet alleen dat ik iets in **as gamta** wil doen."
“Ach, je hebt nog even de tijd om te kiezen! Wel leuk dat je zo van **gamta** houdt!”
“Ja! Ik word altijd vrolijk van **as gamta**.”

“Wat vind je nog meer leuk om te doen Lisa?”
“Ik zit op ballet, want ik houd erg van **sokiai**."
“Cool! Ik heb de Notenkraker wel eens gezien in het theater. Kan je ook op spitzen **sokiai**?”
“Ik heb het wel eens geprobeerd, maar alleen professionele ballerina’s kunnen daar goed op **sokiai**.”
“Als je goed bent in ballet, betekent dat je dan op elk soort muziek goed kan **sokiai**?”
Lisa lachte. "Nee, als je hiphopmuziek opzet kan ik daar echt niet op **sokiai**!”
"En heb jij nog een spannende hobby Daan?”
“Ik heb een **smuikas**.”
“Oh gaaf!”
“Beheers je **as smuikas** goed?”
Daan lachte. “Ik doe het nu al zeven jaar, dus ik beheers **as smuikas** al best goed, al zeg ik het zelf."
“Trouwens, is **smuikas** niet moeilijk te leren?”
“Nou, in het begin vond ik **smuikas** erg ingewikkeld, maar toen ik het eenmaal onder de knie had, viel het mee.”
"Doe je nog andere sporten Lisa?"
“Ja, ik doe aan **ciuozimas**.”
“Dat is bijzonder! Ik ken niemand die doet aan **ciuozimas**. Waar komt die passie vandaan?”
“Mijn vader deed dat vroeger. Hij heeft me geïnspireerd om ook te gaan **ciuozimas**."
“Vandaar! Ben je goed in **ciuozimas**?”
“Redelijk, maar doe het vooral voor de lol. Ik vind het vooral heel leuk om te **ciuozimas**."
“Ben jij eigenlijk sportief, Daan?”
Daan mompelde. “Ik hou niet echt van sporten, maar ik vind mijn conditie wel belangrijk, dus ga ik elke week twee uur baantjes **plaukti**.”
“Dat is toch ook goed! Ik snap wel dat je dan het water in gaat. **Plaukti** is een stuk leuker dan fitnessen of hardlopen.”
Daan knikte. “Anders zou ik het ook niet volhouden! Laat mij maar in het wedstrijdbad **plaukti**, dan ben ik gelukkig.”
“Ik moet je wel een belangrijke vraag stellen Daan. Je gaat toch niet in een Speedo **plaukti** toch?”
Daan schoot in de lach. “Nee, natuurlijk niet! Ik heb alleen een lelijke duikbril op. Ik heb gewoon een normale short aan als ik ga **plaukti**.”

Het eten was inmiddels op. Daan stond op.
“Wil je nog een toetje? Dan haal ik een **ledai** voor je uit de vriezer.”
“Graag! In de zomer eet ik elke dag wel een **ledai**.”
“Wil je een Raketje of een Magnum?" Daan hield beide **ledai** omhoog.
“Een Raketje, alsjeblieft." Lisa wees naar het ene **ledai**.
“Fijn, want ik heb het liefst de Magnum! Dat is mijn favoriete **ledai**!”
“Ik vond het een leuke avond Daan! Moeten we vaker doen!
Maar nu moet ik gaan. Ik moet morgen vroeg op om te werken."
“Oké. Waar werk je?”
“Ik werk bij een **kepejas**.”
“Oh, leuk! Daar kom ik best vaak, maar ik heb je nog nooit bij **as kepejas** gezien.”
“Soms werk ik op een andere locatie. In het centrum hebben ze een veel groter assortiment dan bij onze **kepejas**."
“Ah vandaar. Misschien kom ik je in die **kepejas** dan wel een keertje tegen."
“Lijkt me leuk! Maar nu moet ik echt gaan. Anders ben ik morgen niet fit genoeg en dan zijn ze bij **as kepejas** niet blij met mij.”
“Oké, tot snel Lisa!”
“Doei Daan!”

*Please contact the authors for a copy of the other three story versions.*
